# Monitoring microbial metabolites using an inductively coupled resonance circuit

**DOI:** 10.1038/srep12878

**Published:** 2015-08-12

**Authors:** Daniil Karnaushenko, Larysa Baraban, Dan Ye, Ilke Uguz, Rafael G. Mendes, Mark H. Rümmeli, J. Arjan G. M. de Visser, Oliver G. Schmidt, Gianaurelio Cuniberti, Denys Makarov

**Affiliations:** 1 Institute for Integrative Nanosciences, IFW Dresden, Helmholtzstr. 20, 01069 Dresden, Germany; 2 Institute for Materials Science and Max Bergmann Center of Biomaterials, Dresden University of Technology, Budapesterstr. 27, 01069 Dresden, Germany; 3 Institute for Solid State Research, IFW Dresden, Helmholtzstr. 20, 01069 Dresden, Germany; 4 Laboratory of Genetics, Wageningen University, 6708PB, Wageningen, The Netherlands; 5 Material Systems for Nanoelectronics, Chemnitz University of Technology, Reichenhainer Str. 70, 09107 Chemnitz, Germany; 6 Merge Technologies for Multifunctional Lightweight Structures, Chemnitz University of Technology, Reichenhainer Str. 70, 09107 Chemnitz, Germany; 7 Center for Advancing Electronics Dresden (CfAED), Dresden University of Technology, 01062 Dresden, Germany

## Abstract

We present a new approach to monitor microbial population dynamics in emulsion droplets *via* changes in metabolite composition, using an inductively coupled LC resonance circuit. The signal measured by such resonance detector provides information on the magnetic field interaction with the bacterial culture, which is complementary to the information accessible by other detection means, based on electric field interaction, *i.e.* capacitive or resistive, as well as optical techniques. Several charge-related factors, including pH and ammonia concentrations, were identified as possible contributors to the characteristic of resonance detector profile. The setup enables probing the ionic byproducts of microbial metabolic activity at later stages of cell growth, where conventional optical detection methods have no discriminating power.

High throughput characterization of kinetics of biological and chemical processes including tracking the proliferation of living cells or monitoring enzymatic activity is a key task, particularly, in medical diagnostics, food processing and pharmaceutical industries^1^. The novel bioanalytic approaches using nano- to femto-liter emulsion droplets in fluidics has already surpassed the precision of conventional assays^2^,^3^,^4^,^5^. In particular, the approach has proved itself effective in microbial cell assays for both fundamental microbiology research and clinical studies, *i.e.* to determine metabolic activity, division rate, and level of drug resistance^5^,^6^,^7^.

Analysis of optical signals, *i.e.* photoluminescence^8^ and optical density (OD), is the conventional state-of-the-art method to detect (bio)molecules in multiple-binding assays^9^,^10^, study DNA or RNA abundance in polymerase chain reaction (PCR)^11^ and diverse chemical kinetics^12^, as well as microbial or other cell growth. With the advances in electronics, automated and miniaturized versions of many conventional methods have been developed, such as electronic plate readers for colony counting, or colorimetric or fluorescent assays^13^,^14^,^15^,^16^. Combined with microfluidics, where reagents are used in tiny volumes, these methods gain in performance and precision^7^.

Although methods based on a fluorescent or turbidity signal readout are robust and mature, they have a number of limitations when applied to monitor the growth kinetics of bacterial or cells populations. On one hand, there is the necessity to genetically engineer cells to produce these signals^17^ or to label metabolite molecules produced during cell growth. Another critical limitation of the optical technique is related to the early signal saturation during the measurements. While offering a good limit of detection for OD_600_ (*e.g.* below 10^7 ^cfu mL^−1^ for bacteria), this method reveals the relatively narrow dynamic range of 10–20 dB of measurable analyte concentrations, which requires applying manual dilution of the culture media containing bacteria.

To overcome the limitations of conventional optical approaches, it may be required to develop alternative non-optical detection methods that offer high throughput analyses in a wide dynamic range of microbial dynamics, while remaining cost-efficient, compact and portable. In this respect, new detection strategies for on-chip biosensors based on microelectro-mechanical systems (MEMS) with integrated bio-nanorecognition elements have emerged as a new generation of detectors, allowing species-specific sensing^18^. A promising approach relies on measuring the electrical responses, such as resistance or impedance^19^,^20^,^21^ for monitoring the diverse biochemical characteristics, such as glucose level^22^. Due to the possibility to integrate the sensing elements in fluidic circuitries, the resistive and capacitive detection approaches for milli-, micro- and nano-fluidics are already well developed^23^,^24^,^25^,^26^. However, resistive detection typically involves direct contact between analyzed species and electrodes, which could introduce contamination and difficulties in reusing the system, while impedance-based methods are prone to charge screening due to high ionic strength of liquid analytes. These are sufficiently strong drawbacks limiting applicability of the conventional all-electrical measurement approaches to study biological objects, where monitoring kinetics of living microorganisms or investigating their responses to a microenvironment inherently implies the use of culture media and isotonic buffers. Furthermore, capacitive sensors require external bulky and expensive impedance analyzers, which are not in the spirit of the compactness and portability offered by the lab-on-chip concept.

Here, we present the millifluidic resonance detector (MRD), consisting of an inductive coil wrapped around a capillary tube and employ it for analysis of water-in-oil emulsion droplets containing bacteria (Fig. 1a,b). In contrast to the previously proposed capacitive platforms, the detection schema of the MRD is based on the inductive coil, which is placed around the channel and produces a uniform alternating magnetic field within a channel. Considering the size of the coil of about 1 mm and the working frequency of 2 MHz with the corresponding wavelength of 150 m in vacuum, accompanied with the fact that the electric field is locked between the windings of the coil, the linear dimensions of the coil are too small to produce any electric field in the channel. Therefore, the coil is coupled purely *magnetically* to the content of the droplets. In this respect, the proposed detection approach provides information on the magnetic field interaction with the analyte, which is complementary to the information provided by other detection means based on the electric field interaction obtained by capacitive, resistive and optical approaches. Due to this specificity, the device opens new opportunities to assess the ionic byproducts as a result of bacterial growth in emulsion droplets and provides the possibility to monitor the metabolic activity of the bacteria in the single experiment without intervention.

## Results

### Millifluidic resonance detector

**Figure 1:**
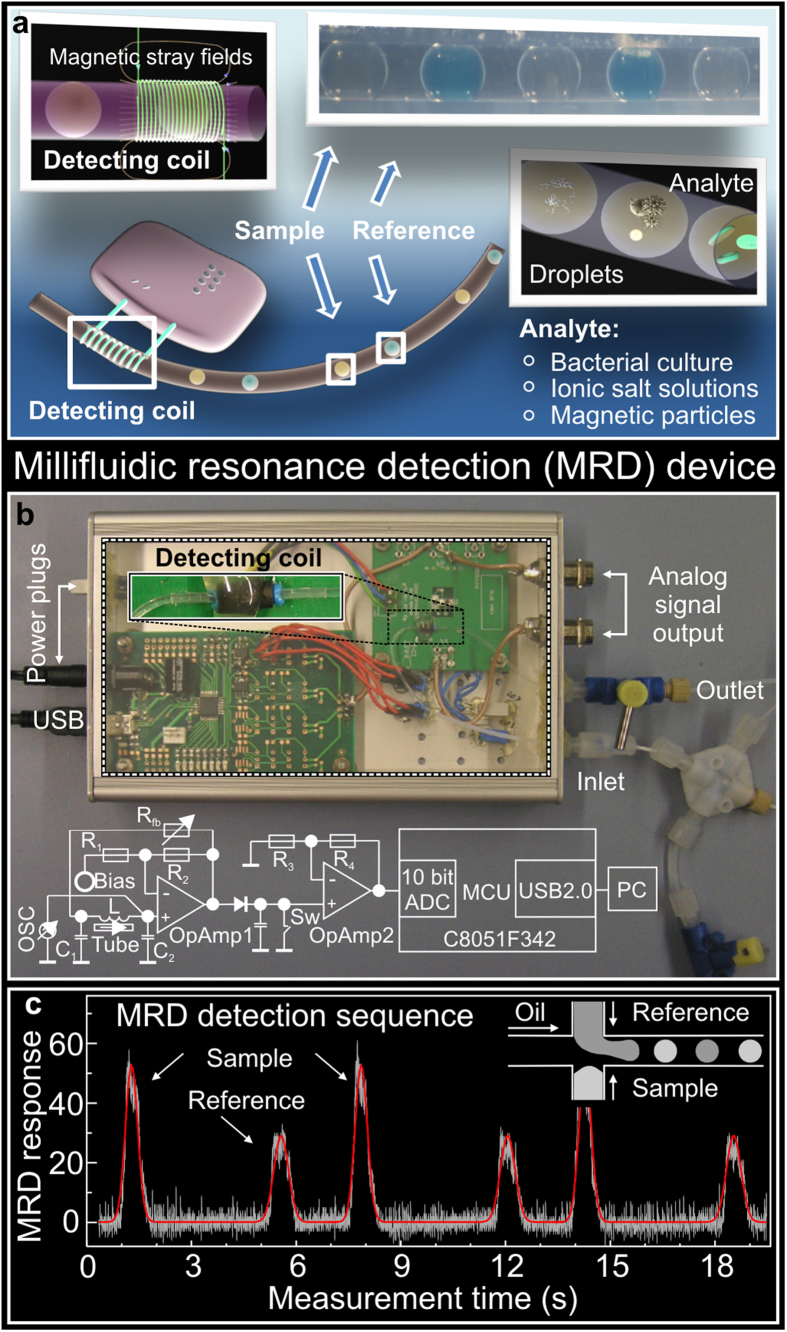
Millifluidic resonance detector (MRD): (**a**) Schematics of the setup, showing the inductive coil wrapped around the fluidic channel containing droplets. Alternating aqueous droplets of analyte and reference (*i.e.* bacterial culture, magnetic nanoparticles) pass through the interior of the inductive coil one by one, each registering a signal peak – the MRD response. (**b**) Optical micrograph of the detector unit assembled in a commercially available case. The electronic circuitry as well as the detecting coil are shown. (**c**) The MRD response of the chain of aqueous analyte droplets containing superparamagnetic Fe_3_O_4_ nanoparticles (sample) separated by the droplets containing DI water (reference). Alternating chain of emulsion droplets is formed using a cross-junction geometry (inset). Red solid line in panel (**c**) is a Gaussian fit to the data, which was used to analyse the amplitude and full width at half maximum of the individual peaks.

The main challenge behind realization of the inductive detection approach for fluidics is the need to combine it with an extremely sensitive, but portable and cost-efficient detector, which enables to measure the variations of the inductive part of the two port impedance coupled to the droplet-based fluidic system. We overcome this hurdle by combining the inductive coil and external capacitors into an LC resonance tank, which is inserted into the Colpitts oscillator scheme to form an autodyne (Fig. 1b; see also the Supplementary Fig. 1). The resonance frequency of the circuit depends on the total capacitance and inductance connected in parallel, resulting in a parallel resonance circuit. Hence, the impedance of the LC tank at the resonance frequency is high and out of resonance is low. The circuit is realized in a way that the resonance frequency will vary only with variation in inductance and not with variation in capacitance (Supplementary Information). Inductance can vary only with respect to the total permeability defined by the coil core represented by the tube with the analyte. Hence, the permeability is affected by the magnetic analytes or alternatively by the induced magnetic field produced due to eddy currents in an ionic liquid.

The LC circuit is excited by the constant amplitude AC current source at constant frequency, producing an AC voltage signal. The excitation frequency is selected at the region of the highest slope of the resonance peak corresponding to the maximum sensitivity region of the device. As a result, by monitoring changes of the amplitude and/or phase of the measured voltage, we see shifts of the resonance frequency. The analogue output of the detector is AC voltage, which is then digitized using a 10-bit analog-to-digital converter (ADC). The digitized signal is named “MRD response” and is presented in ADC units, which are fractions of the full range of the 10-bit ADC equal to 1024 ADC units. For instance, the maximum MRD response of about 60 ADC units is shown in Fig. 1c. The MRD response can acquire any value depending on the excitation frequency, chosen electronic components and the experimental conditions such as temperature. Therefore, the ratio between the amplitudes of the MRD responses of the analyte and reference droplets is used (named in the following the “MRD signal”) as a representation of the physico-chemical properties of the content of the droplet. This measurement protocol provides access to the change of the signal with respect to the reference substance and allows compensating for the possible signal drifts. The steeper the slope of the resonance peak of the detector, the larger the change will be measured (higher device sensitivity). To achieve steeper slope, we realized a regenerative circuit connected to the LC tank which compensate losses and hence enhances the quality factor, slope and therefore sensitivity of the device.

At the operation frequency of the resonance circuit of about 2 MHz and the experimentally determined full width at half maximum of the resonance peak of about 20 Hz (Supplementary Fig. 4), the quality factor (=the ratio of the operation frequency to the full width at half maximum of the resonance peak) of 10^5^ is achieved without the use of bulky external equipment. Hence, the sensitivity of the developed electrical resonance circuit integrated in the millifluidic platform is comparable to the performance of advanced sensorics employing the high quality optical resonators^27^,^28^ or MEMS^29^,^30^,^31^,^32^.

With the sensitivity of about 10 dB/Hz, the MRD device measures tiny shifts in resonance frequency as a result of changes in the coil’s inductance once continuous oil phase and aqueous droplets with encapsulated biological species (analyte) alternate when flowing through the core of the coil. The sensitivity is determined as the highest slope of the resonance peak and calculated as the change of the output signal of the detector (in log scale) per Hz. The respective data is shown in Supplementary Fig. 4. Dynamic range of the device is limited to about 26 dB by the noise of the electronic components and the 10 bit ADC. The dynamic range of the MRD is calculated as follows: The average noise level of the MRD response (raw data from the detector) is 2.6 ADC units. Accounting for the full range of the 10 bit ADC (=1024 ADC units), the noise in the log scale is 10xlog(1024/2.6) = 26 dB. This value is further reduced down to about 14 dB accounting for the linear part of a resonance slope (about ½ of the initial ADC range). This is comparable to the dynamic range of 10–20 dB of the conventional optical devices applied for turbidity (optical density, OD) measurements.

**Figure 2:**
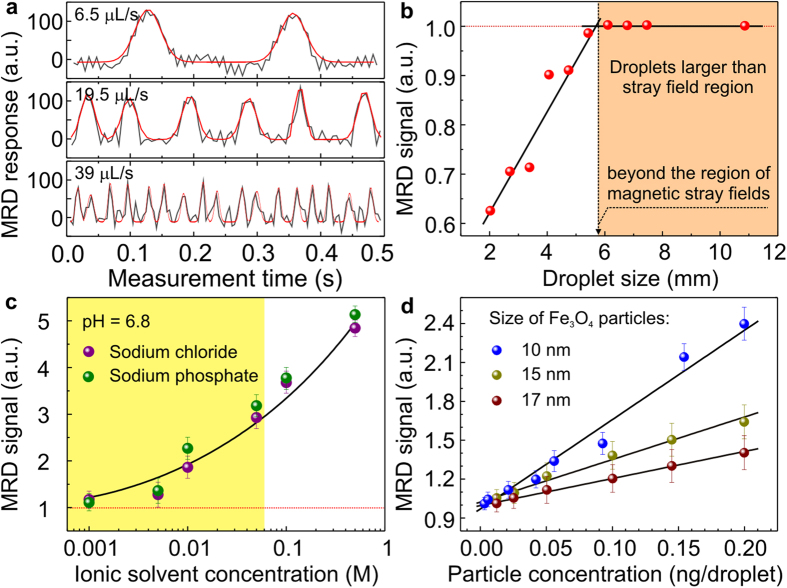
(**a**) MRD response *vs.* time at different flow rates. Red solid lines in panel (**a**) is a Gaussian fit to the data, which was used to analyse the amplitude and full width at half maximum of the individual peaks. (**b**) MRD signal (=ratio between the amplitudes of the MRD response of the analyte and the adjacent reference droplet) *vs.* the droplet size. Orange region in (**b**) designates the droplet sizes, equal or larger than the region of the magnetic stray fields. (**c**) MRD signal as a function of the sodium chloride and sodium phosphate concentration. Region indicated in yellow in (**c**) corresponds to the physiological range (ionic strength, characteristic for the growth medium). (**d**) The measured MRD signal linearly increases with the concentration of magnetic nanoparticles. The effect of the particle sizes is shown as well.

The fluidic circuit of the MRD is capable of generating water-in-oil emulsion droplets using a cross-junction geometry and transferring them towards a detection area through a thin capillary tube with an inner diameter of 250 μm (inset in Fig. 1c and Supplementary Information). Microfluidic pumps were programmed to produce reference and analyte droplets one by one in order to minimize possible cross-contaminations between the neighboring droplets. The MRD platform can perform detection of water-in-oil emulsion droplets at the flow rate up to 39 μL s^−1^ corresponding to the droplets rate of 35 Hz (Fig. 2a; details are given in Supplementary Fig. 6). With this performance, the MRD can be applied for high-throughput screening in fluidics allowing to analyse a 1 mL solution within less than 10 min. The achieved screening speed is limited by the fluidic platform and the emulsion stability but not by the detector itself. The 10 bit ADC with the maximum frequency of 200 kHz is operating at 800 Hz and can detect safely up to 100 droplets per second, with about 5 points per droplet. In this operation mode, the MRD can be applied as an independent droplet counter of the non-transparent fluidic circuit allowing to monitor the droplets rate in a channel (Supplementary Fig. 3). This feature allows to extend the capabilities of a fluidic platform towards chemical reactions with direct involvement of light-sensitive molecules, *e.g*. dynamic combinatorial libraries for hormones analysis^33^ aiming to avoid their degradation.

**Figure 3:**
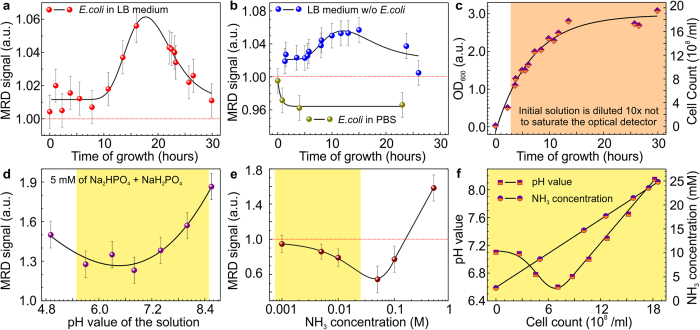
Monitoring metabolites of the *E.coli* population: (**a**) MRD signal of *E.coli* batch culture in LB broth. Fresh LB broth was used to form the reference droplets. The solid red line is a guide to the eye. (**b**) MRD signal of *E.coli* cells isolated from the culture medium and resuspended in PBS buffer (dark yellow symbols). MRD signal of *E.coli* batch culture in LB broth after cells have been removed (blue symbols). The time shift of the peak of MRD signal in panels (**a**) and (**b**) is due to differences in conditions in the bacterial cultures used for the bacterial measurements (**a**) and medium tests (**b**). Fresh LB broth was used to form the reference droplets. The solid line is a guide to the eye. (**c**) Growth kinetics of the bacteria cultured in a flask and monitored using conventional measurements of optical density OD_600_. White region in (**c**) corresponds to the OD_600_ measurements before the sample dilution was performed for the first time. The dynamic range of the turbidity measurement of the present setup is about 0.5, corresponding to about 10 dB. Orange region corresponds to the measurements, when batch culture was 10x diluted. (**d**) The MRD response as a result of pH variation. Measurements are done at constant ionic strength of the solution (5 mM sodium phosphate). (**e**) The MRD signal *vs.* the ammonia concentrations. (**f**) Reference measurements, determining evolution of pH value and NH_3_ concentrations during growth of bacteria. Yellow region in panels (**d–f**) indicates the range of conditions, close to physiological (compatible with bacterial growth).

In the following experiments, the flow rate will be kept constant at 0.25 μL s^−1^. The MRD signal amplitude increases with the increase of droplet size but saturates after the size reaches about 6 mm, which is approximately the size of the effective stray field region of the coil (orange region in Fig. 2b). The signal readout depends not only on the size but also on the content of the emulsion droplets. The capabilities of the device to address tasks in analytical chemistry and micro- and cell biology are demonstrated using the strain MC4100-YFP of the bacterium *Escherichia coli*^17^. In order to monitor metabolites due to the metabolic activity of *E.coli* populations over time, the physical, chemical and biological quantities measured by the MRD have to be addressed properly taking into account the resonance circuit specificity and the complexity of the culture medium. Bacterial growth will cause changes in the type and amount of biochemical species in each droplet. This includes the depletion of carbon and nitrogen sources as well as the accumulation of metabolic byproducts such as carbon dioxide, acetate (electrically charged), and ammonium (electrically charged). Hence, bacterial growth results in the modification of the medium affecting, among others, its ionic strengths and pH (these parameters are related). For instance, growth of *E.coli* populations initially leads to a decrease of pH of the medium from neutral to slightly acidic. At later stages (mid-exponential phase), the pH of the culture increases towards alkaline values (Fig. 3f). Another important parameter is the ammonium concentration, which reveals a linear increase during the bacterial growth process (Fig. 3f).

To extract the contribution of the bacteria to the MRD signal, we first calibrate the MRD platform using conventional optical and chemical measurements with respect to the parameters of interest. This means that we perform experiments with different reference analytes, including high dissociation salt solutions (Fig. 2c and Supplementary Fig. 7) at various ionic strengths, magnetic nanoparticles (Fig. 2d and Supplementary Fig. 11–13), pH values (Fig. 3d and Supplementary Fig. 9), ammonium (Fig. 3e and Supplementary Fig. 15) and acetate (Supplementary Fig. 16) concentrations. The calibration is carried out by comparing the MRD signal with the data acquired using conventional methods, such as OD, pH, ammonium/acetate/glucose kits, chromatography, and cell counts.

The sodium chloride and sodium phosphate were chosen for investigation as these substances are typical constituents of the bacteria growth medium, helping to maintain its isotonic balance. Aqueous solutions of salts were measured for their MRD responses, with deionized water as the reference. The MRD signal is proportional to the salt concentration for concentrations of up to about 0.05 M and is the same within the error bars for sodium chloride and sodium phosphate solutions (Supplementary Fig. 7). Considering the proportionality between the concentration of fully dissociable salt solution and its conductivity, this observation indicates proportionality between the power and the conductivity similar to the case of eddy current losses. Hence, the MRD measures the power loss due to induced eddy currents generated within the droplet containing conductive salt solution which is proportional to the ionic strength, or more generally, the conductivity of the droplet content. To cross-check this assumption, we carried out additional tests on glucose as an example of non-ionic and uncharged molecules, which is a standard and dynamically changing component of the growth medium. The MRD signal remained around 1, showing little difference within each analyte-reference droplet pair and across different glucose concentrations (Supplementary Fig. 8).

When detecting magnetic species (Fig. 2d), the MRD response is proportional to the total magnetic moment of the droplet material. For these experiments, two different suspensions of magnetic Fe_3_O_4_ nanoparticles (9–20 nm diameter)^34^ as well as Co nanoparticles (50 nm diameter) were homogenized prior the droplets formation. The detection limit for the superparamagnetic Fe_3_O_4_ nanoparticles of ~10 μg mL^−1^ is achieved (Supplementary Fig. 10–13), which lies in the range typically employed for laboratory cell toxicity tests, *e.g*. for tumor cells investigations. Based on these calibration results and keeping in mind that *E.coli* possesses low magnetic permeability and poor electrical conductivity, we conclude that the cells themselves are not expected to impact the MRD signal. In summary, the data presented in Figs 2c and 3 accompanied with careful calibration of the device suggest that the measured MRD signal from the bacterial culture is related to changes in the conductivity of the droplet content due to metabolic activity of the cells during growth.

### Bacterial measurements

With the previously obtained information, we apply the MRD for tracking the metabolic activity of *E.coli* strain MC4100-YFP in LB broth. Growth of the culture comes with changes in cell concentration and associated changes in the type and amount of metabolites^16^. This includes the depletion of carbon and nitrogen sources, as well as the accumulation of metabolic byproducts such as carbon dioxide, organic acids and ammonium. A batch culture was set up in a thermostatic incubator and was automatically sampled by the millifluidic inlets of the MRD every one to two hours, recording up to 150 droplets per measurement. Sampling of the droplets from the external reservoir was realized in order to ensure aerobic respiration of the *E.coli* cells during the whole experiment. Measurements of the optical density at a wavelength of 600 nm (OD_600_) using a spectrophotometer were performed in parallel, as a reference (Fig. 3c, Supplementary Fig. 14). It has to be noted that the 10-fold dilution of the batch culture from the reservoir, after the OD_600_ reached 0.5 (log scale; about 3 hours of incubation), was realized in order to avoid signal saturation due to the strong absorption of the light by the optically dense medium. Transition from white to orange in Fig. 3c indicates when the first dilution was performed.

A clear reproducible change in the MRD signal from the droplets containing bacteria during growth was observed (cf. Fig. 3a and Fig. 3c). The signal increased from 1 at time zero to about 1.055 after 15–20 hours, around the time of transition from exponential to stationary phase (where the cells stop growing), before it decreased to about 1.02 later in stationary phase. As cells generally have low magnetic permeability and poor electrical conductivity, they were not expected to influence the electromagnetic field in the coil. This assumption was checked by resuspending bacterial cells into PBS buffer every hour and by corresponding measurements with the MRD. Interestingly, measuring the culture medium with cells removed (Fig. 3b) shows a trend similar to that shown in Fig. 3a. We performed three control measurements of the pure medium (cultured nearby in an incubator) at times *t*_1 _= 0 h; *t*_2 _= 3 h, *t*_3 _= 24 h. The study was carried out to exclude the possible medium contamination. The MRD signal for these control measurements was found to be in the range of 0.99–1.008. These changes are substantially smaller than the detected signals of droplets with cells. It was thus concluded that changes in the MRD signal are mainly a result of the changing composition of the medium during culturing, rather than the increasing number of bacterial cells, which is the target of measurement in conventional growth characterization methods. Therefore, the specific attention to the production of metabolites during bacterial growth should be paid.

The major path of energy generation in aerobic mode of *E.coli* and all other aerobic organisms is through the citric acid cycle. The total amount of carbon dioxide, acetate, and ammonium produced can thus indicate the state of growth. For instance, acetate production is often observed in cultures of *E.coli* when the carbon flux into cells exceeds the capacity of the citric acid cycle to consume acetyl-CoA^35^. In media offering proteins and peptides as the major carbon and energy source, *e.g*. LB broth^36^, the deamination of amino acids prior to entering the citric acid cycle produces ammonium, the amount of which exceeds the cells’ need for nitrogen. This excess amount of ammonium excreted into the culture medium can be tracked and related to bacterial growth in batch culture^37^. Further and inter-reactions of metabolites may cause their concentration along the course of growth to follow a non-monotonic trend, rendering the one-to-one correspondence between the metabolite amount and the cell concentration impossible. For example, excess acetate, which is first excreted by the aerobic *E.coli,* is later re-assimilated. The pH value of the culture may also experience both increases and decreases in the same growth cycle due to the interplay between the acidic and alkaline metabolites excreted into the medium. Several other common waste products produced by *E.coli* include lactate, ethanol and succinate, but they are often the result of anaerobic respiration and do not help to characterize population dynamics during aerobic growth. Therefore, we do not address these latter chemical parameters. Thus, several charge-related factors, including pH, ammonia, acetate concentrations, were identified as possible contributors of the MRD signal and tested using reference techniques (details in Supplementary Section 3).

## Discussion

From the multiple reference measurements, we propose that the increase in MRD signal after about 10 hours in the exponential phase is attributed to an increase of pH of the culture medium from physiological level up to pH = 8.5 (Fig. 3d). As the *E.coli* populations grew, the pH value of the medium changed non-monotonously with an increase from 6.5 to greater than 8.5 (after ~6 hours) (Fig. 3f, square symbols). Therefore, the MRD allowed to detect an *onset process*, when the pH value of the medium turns from the neutral into the alkaline. The further decrease of the MRD signal in stationary phase (after ~22 hours) may be attributed to an increase of the ammonium concentration associated with an increase in the number of cells and resulting assimilation of proteins (present as tryptone in the medium) as a carbon source (Supplementary Fig. 15).

Tests with aqueous ammonia solutions revealed that an increase of ammonium concentration within the physiological concentration range up to 25 mM of a bacterial culture led to a decrease of the MRD signal despite the increase in ionic strength and pH (Fig. 3e,f). In this respect, it is suggested that ammonium at a concentration below a certain critical may be able to capture residual ions present in water, which is not fully de-ionized. Yellow regions in Fig. 3d–f indicate the physiological range of the measured substances during bacterial growth under the culture conditions applied. To probe the MRD signal of the acetate, the acetic acid aqueous solution in the concentration range 0.1 mM to 0.5 M was prepared and used as the analyte. The deionized water was used as the reference. Furthermore, the amount of acetate in the growing culture was evaluated by gas chromatography with a polyethylene glycol-capillary column (Supplementary Fig. 16a,b). The MRD signal taken of the acetate droplets with the different acetate concentrations did not reveal a strong change within the entire physiological range (Supplementary Fig. 15b). Most probably this is due to the low dissociation capability of the acetate compared to the other substances carrying higher electrolytic properties (high dissociation coefficient).

The MRD signal of the growing *E.coli* culture in LB broth showed reproducible variation, revealing a signal increase in the late exponential phase (10–15 hours) and a decrease in the stationary phase (15–30 hours). This finding is confirmed by measuring the MRD signal of *E.coli* cultured in synthetic minimal salts medium M9.

In conclusion, we proposed a novel inductively coupled resonance detector integrated into a fluidic channel for monitoring microbial metabolism kinetics of bacterial growth in emulsion droplets. We demonstrate that in contrast to the conventional growth characterization using optical methods, which measure the cell concentration directly and is capable of tracking some of the metabolic parameters, the MRD signal of a bacterial culture is mainly attributed to the change of medium composition along the course of growth. We propose that the charge-related factors such as pH changes and changes in ammonium concentration during the growth are likely to be responsible for the measured MRD signal. Hence, the MRD analysis of the ionic byproducts of the culture media enables to probe metabolic activity of the population at the later stages of growth.

This is the very first realization of the sensoric system for fluidics which access the information based on the magnetic field interaction with the analyte. Accordingly to the presented data, when once calibrated using conventional optical and chemical measurements with respect to the parameters of interest, the MRD device could be readily used even for quantitative investigations of the complex analytes. Still, it is important to stress that independent calibration is needed for different types of bacteria, possessing different metabolic paths.

The unique feature to collect the information based on the magnetic field interaction with the analyte offers numerous advantages over the state of the art approaches:

- Remarkable sensitivity of 185 dB M^−1^ to the ionic strength of solution, which is superior over the optical methods (see Methods section for further details);

- Possibility to perform label-free measurement of pH;
- No need of transparent packaging as for optical measurements. This option might be of advantage to study light-sensitive analytes;
- The possibility to monitor the metabolic activity of the bacteria in the single experiment without any human intervention. The latter is not possible using other approaches;
- The MRD can capture the onset when the medium starts to be affected by the increased ammonium content. This information is not accessible using the state of the art fluidic approaches.

These benefits suggest that the realized MRD platform can serve as a tool for analytical chemistry enabling to monitor kinetics of several chemical reactions, accompanied with pH, ionic or magnetic content change, contactless and in-flow. Since different groups of cells reveal different metabolic pathways, this technique might offer certain cell specificity. Finally, we envision great potential to extend the sensing capabilities of the MRD to realize automated analytic tools, which could be of advantage for (*i*) monitoring cellular growth inside of isolated emulsion droplets, (*ii*) to realize a chemostat enabling controlled continuous feeding of the growing culture, or (*iii*) determining and isolation of the pathogenic strains *via* integrating selection module. At the same time, it is important to emphasize that at the present stage of its development, the MRD platform is not able to detect specifically chemical substances appearing during bacterial/cells growth. Therefore, in this work we monitor the generalized picture of the population growth, which is reflected in the MRD signal and represents a fingerprint of certain bacteria growing in a certain media.

In this respect, to allow for the quantitative analysis of specific metabolites, the MRD platform should be combined with other available detectors, *e.g*. optical sensors (in particular, based on fluorescence) for specific assays, like detection of oxygen or pH. We envision that by extending the present platform towards spectroscopic in-flow measurement protocol should provide the highly demanded specificity to the MRD device.

## Methods

### Resonance detection module

The MRD consists of an inductive coil wound around a fluidic channel and external capacitors, thus forming an LC resonant tank circuit inserted into the Colpitts oscillator scheme to form an autodyne (Supplementary Fig. 1). The quality factor of such a resonator can be enhanced dramatically when the feedback compensates energy loses and the circuit goes into oscillation on its fundamental resonance frequency, which is defined by inductance of the coil and capacitances of capacitors included in the circuit (Supplementary Fig. 1 and 3). If the magnetic permeability of the core of the coil is modified by induction currents or magnetic material, inductance of the coil will change influencing the resonance frequency. Here, we detect the variation of the resonance frequency by monitoring the changes of the amplitude at the defined frequency close to the fundamental resonance of the LC tank. In order to lock the circuit to oscillate at the defined frequency and phase, the feedback signal has to be reduced to the value, which is sufficient to suppress the fundamental oscillation, and a current from an external oscillator has to be fed into the LC tank (Supplementary Fig. 1). The magnitude of the current was chosen to initiate oscillations of the circuit, phase and frequency locked to the external oscillator. The amplitude and the phase of the signal at the output of the circuit vary with the frequency of the resonance peak. By tuning the frequency of the external oscillator, the working point can be set to be either on the left or right slope of the resonance peak. In our device, due to the properties of the coil, the lower frequency slope has higher sensitivity. By tuning the feedback signal, the quality factor can be enhanced to values approaching 10^5^ thus providing sharp slopes and a high sensitivity. This quality factor of the fabricated LC circuit approaches values achieved in MEMS raising the possibility to analyze low concentrations of ferromagnetic, superparamagnetic particles and ionic liquids.

The MRD provides the information on the *magnetic* field interaction with the analyte, which is complementary to the information provided by other detection means based on the *electric* field interaction obtained by capacitive, resistive and optical approaches. This unique feature offers numerous advantages in terms of sensitivity to the ionic strength of solutions, label-free measurement of pH and especially provides the possibility to monitor the metabolic activity of the bacteria in the single experiment, potentially without any human intervention.

Along with the advantages of the novel method highlighted above, we indicate its limitations:

- Absence of chemical sensitivity. Impedance on-chip spectrometry should be realized to equip the MRD with the chemical sensitivity;
- Need of careful calibration using optical or other electric-based approaches to assess quantitative information (see details on calibration below).

#### Design of the device

An inductive coil with about 70 windings of an isolated copper wire of 100 μm in diameter is winded around a glass tube with an outer diameter of 1 mm. The coil has a width of about 1 mm; effective magnetic field spreading out of the coils is estimated to be about 6 mm. An FEP (fluorinated ethylene propylene) capillary tubing of 0.8 mm in outer diameter and 0.35 mm inner diameter is inserted into the glass tube. The coil is soldered to a printed circuit board consisting of zero temperature coefficient ceramic capacitors of 10 nF (resulting in a resonant frequency of ~2 MHz) and an operation amplifier OPA602 with a tuned feedback to the LC tank. The board contains also a peak detector and a USB 2.0 microcontroller C8051F342 having built-in analog to digital converter (ADC) (Supplementary Fig. 1 and 2). To excite the circuit, an external function generator (in the present work: Tektronix AFG3101) has been used.

The feedback trimming is accomplished by two resistances in series 100 Ohm for fine and 10 kOhm for rough adjustment and represented as a single R_fb_ (Supplementary Fig. 1). The excitation signal from the functional generator of about 1 V in amplitude is applied to the LC tank through the 100 kOhm resistor, which is shown as an OSC in Supplementary Fig. 1. The first cascade around the operational amplifier (Op. Amp. 1) is biased to about 0.7 V to overcome the diode threshold of the peak detector. Then the signal is integrated for about 10 to 20 periods on capacitance directly after the rectifying diode, amplified by a cascade around Op. Amp. 2 and acquired by a 10 bit ADC integrated into C8051F342 microcontroller. Resistances R_1_ and R_2_ around Op. Amp. 2 assemble a potentiometer of 10 kOhm, which is used to adjust the amplification of the signal from Op. Amp. 1. After completion of the acquisition circle the peak detector is discharged by closing the switch SW for several μs. The number of measured points is collected in the microcontroller and then sent *via* USB 2.0 interface to host computer for analysis.

The relative temporal variation in amplitude for a specific frequency represents the measured signal (Supplementary Fig. 4). The excitation frequency is tuned to be settled close to resonant peak on the low frequency slope as well as feedback coefficient is also tuned to give a quality factor of around 10^5^ and a sensitivity of about 10 dB/Hz. High sensitivity requires particular attention to stabilization of ambient conditions which had been paid especially for temperature of the coil, varying slightly inductance and shifting constantly working point. Therefore the system was warmed up prior to experiments for about 30 min with a flowing emulsion through the tube. High throughput of the fluidic system and detection speed brings an advantage of differential characterization, thus simplifying data acquisition, their analysis and making contribution of the disturbances such as temperature variation to useful signal negligible.

### Fluidic module

Water-in-oil emulsion droplets were produced in a cross-junction geometry and formed a one-dimensional train. Water droplets were dispersed in hydrofluoroether oil (HFE-7500, 3M) and stabilized by 0.02% PicoSurf surfactant is produced in a capillary tubing (fluorinated ethylene propylene, FEP). To account for the random drifts of the resonance detector, a reference droplet (solvent of the analyte droplet) is placed next to every analyte droplet, so that the signal ratio between the analyte and the adjacent reference droplet can be recorded as a more accurate measure of the detector response (Supplementary Fig. 5). To minimize cross-contamination between the two different contents, droplets are produced by pumping the analyte, the oil spacer and the reference sequentially through the cross-junction instead of simultaneous injection which was the standard way of generating droplets in the squeezing regime.

Although the device is able to accommodate a droplet flow rate of up to 39 μL s^−1^ and to detect up to 30–40 droplets per second (Supplementary Fig. 6 and Fig. 2a of the manuscript), all experiments are carried out at constant flow rate of 0.25 μL s^−1^. The size of the droplet was maintained consistently to be larger than the length of the region of the magnetic stray fields (6 mm in the present experiment), at about 20 mm, so that small variations in droplet size do not affect the signal ratio (Supplementary Fig. 6). Due to the high throughput of the MRD, more than 20 droplet pairs were measured for each data point. The error was calculated as the standard deviation of the signal ratios across these droplet pairs. A MatLab code was developed and applied to filter the raw data and fit the signal peaks with square step functions. The height of each peak could be calculated as the difference between its upper plateau section and the adjacent baseline section. In this way, the demanding task of fitting an overall baseline was bypassed.

#### Cross-contamination between the droplets

The following two aspects were taken into account to avoid the cross-contamination between the neighboring droplets:

#### Use of surfactant

We employed “one-by-one” droplet formation technique, where the injection of reference and analyte liquids is performed not in the continuous fluid flow, but using pulsed pumping regime, working in counter phase. Injection conditions were selected such that the MRD signal ratio between reference and analyte droplets is maximized (tests were performed using salt solutions as the analyte and deionized water as the reference).

#### Diffusion of the components through the oil phase

This artifact is practically excluded, since the droplets sampling and measurements are performed in about 5 min, at longest, which is too short time for the diffusion of the components through the water/oil interface and oily phase.

### Dynamic range of the MRD

The dynamic range of the MRD is limited by the noise of the electronic components and the 10 bit ADC to a value of about 26 dB. Considering the linear part of the resonance slope, the dynamic range is further limited to about 14 dB.

### Calibration of the MRD for quantitative measurements of the analytes

The quantitative data analysis using the MRD platform requires reference information. This can be achieved in the two-step process:

1. The MRD should be always applied by measuring the material of interest dispersed in a solution with respect to the solution. Then, the measured MRD response (ratio between the two signals) over time will reflect either the evolution of the material itself in this solution (*e.g.* agglomeration of magnetic nanoparticles with time) or the change of the solution due to the impact from the material (*e.g.* the metabolism studies).
2. The interpretation of the results of the physical, chemical or biological origin behind these measured changes should be carried out based on the carefully prepared calibration curves which are obtained by using the MRD platform in conjunction with the optical or electrical methods. These curves are obtained by measuring the MRD signal change with respect to the parameters of interest (optical density, temperature, pH, salts, glucose, and ammonium).

When once calibrated using appropriate conventional optical or electrical measurements of the parameters of interest, the MRD device could be readily used even for quantitative investigations of the complex analytes. One of the main goals of this manuscript is to provide such calibrations for the novel MRD platform.

### Sensitivity of the MRD to different analytes

*Determination of salt concentrations:* The MRD reveals a linear response to the change of the sodium chloride or sodium phosphate concentrations until 0.05 M (about 0.003 g mL^−1^) giving a signal response of about 9 dB / (0.003 g mL^−1^) ≈ 3180/(g mL^−1^) or 185 dB M^−1^. The signal ratio equals 1 for the DI water and 3 for 0.05 M of NaCl. In Fig. 3a of Erickson *et al.*^27^ the transmission spectra of a photonic crystal is shifted by changing the solution from H_2_O to 5 M CaCl_2_ in H_2_O. Taking the slope starting at 0.285a/λ (normalized transmitted power = 0 dB) and by immersing the resonator in a 5 M of CaCl_2_ solution, the slope is shifted giving 20 dB variation in the signal which corresponds to 4 dB M^−1^.

Another example is described by Song *et al.*^28^, where the micro-machined microtoroid optical resonator was coupled to a fluidic system and was subjected to various solution concentrations of NaCl. The ability of the optical resonator to detect small concentrations of the salt in water is clearly indicated in Fig. 5b-5c of the reference 28, where the wavelength shift of 0.125 nm is observed when the concentration of NaCl is changed for 0.005 g mL^−1^. Taking into account the Q-factor of the resonator of 3.2 × 10^3^, the shift of a such sharp peak to 0.125 nm will correspond to the variation of 0.257 (=3200 × 0.125 nm/1557.4 nm) in the normalized transmittance. The signal then about 2 to 3 dB per 0.005 g mL^−1^, which is 600 dB/(g/mL) or 35 dB M^−1^.

This comparison indicates that the MRD platform is superior over the optical detection methods in identification of the salt concentration.

We admit that complex impedance measurements could further improve the performance of the MRD. For instance, Luo *et al.*^24^ realized the complex impedance sensor measuring resistance and capacitance. In this case, they can get signal of about 18 dB in the linear regime at 500 kHz by measuring the NaCl concentration up to about 0.01 M leading to the device sensitivity of about 1400 dB M^−1^.

#### pH measurements

It is important to emphasize that all optical methods rely on the detection of dye luminescence, which are pH sensitive (such as Seminaphtharhodafluorescein used by Seemann *et al.*^38^ and Magnusson *et al.*^39^). Without using dyes, pH tests are not possible to be carried out optically. This labelling requires human intervention to the analyte, which is often not desirable and should be avoided.

With the dye, the pH sensitivity down to 0.05–0.1 can be achieved^38^ with the linear wavelength shift with respect to pH starting from pH about 6.5 to about 8.5. In this work^38^, the measurement of the sample with a volume of 1 nL for 20 min was required.

In contrast, the MRD platform does not require labelling to perform the pH measurements. In the range of pH from about 6.5 to about 8.5, the MRD has linear response and by noise limited precision to about 0.2. Our device is able to measure 1 μL droplets per 0.03–0.04 s. Assuming that the optical signal is linearly proportional to sample volume, the pH value of the 1 μL sample has to be measured optically with the precision of 0.05 for about 1.2 s. From the standard shot noise considerations, the decrease of the acquisition time by 16 times would result in the increase of the error bars 4 time to about 0.2 to measure optically the sample with the volume of 1 μL within 0.075 s^40^. This is about twice worse compared to the performance of the MRD platform.

### Cell counting

With the low limit of detection (signal is obtained only after about 8 hours of the bacteria growth), the MRD cannot be positioned as a platform for cell counting. Nevertheless, in the period of time from 8 to about 16 hours, where the MRD has linear response (Fig. 3a), the signal variation for cell growth using OD_600_ taking into account the 10x dilution of the medium is only slightly higher (0.07 (log scale) or 1.4 dB for OD_600_), compared to 0.05 (log scale) or 1 dB for the MRD.

### Detection of *non-magnetic* objects

#### Ionic strength and conductivity measurements

The performance of the MRD to detect non-magnetic conductive objects was tested on fully dissociable salts (sodium chloride and sodium phosphates) as presented in Supplementary Fig. 7. Aqueous solutions of fully dissociable salts were measured for their MRD responses, with deionized water as the reference. The MRD signal increases from 1 to 5 as the concentration of NaCl increases from 0 to 0.5 M. Plotting the MRD signal versus the salt concentration, *c*, yielded a linear dependence for concentrations of up to about 0.05 M (Fig. 2c and Supplementary Fig. 7):
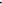



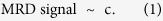


Considering the proportionality between the concentration of fully dissociable salt solution and its conductivity, the relation depicted in Eq. (1) indicates the proportionality between the power and the conductivity. Since the interior of the coil experiences an alternating magnetic field due to the applied AC current, eddy currents are induced within the droplet containing conductive salt solution passing the core of the coil. These eddy currents in turn induce magnetic fields that oppose changes in the current, thus changing the inductance of the coil and causing a shift in frequency of the resonance circuit. The greater the conductivity of the droplet content, the greater the eddy currents and the magnetic fields induced, leading to greater power loss and a bigger change in the inductance. It is known that the power loss (per unit mass) due to eddy current is proportional to the conductivity of the material:


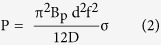


where 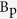 is the amplitude of the magnetic field, 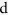 is the diameter of the droplet, 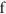 is the frequency, and 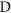 is the density of the material. This is consistent with the obtained proportionality between power and the conductivity. We therefore postulate that for non-magnetic conductive droplet contents, MRD measures the power loss due to induced eddy currents which is proportional to the ionic strength, or more generally, the conductivity of the droplet content.

To cross-check this assumption, we carried out additional tests on glucose as an example of non-ionic and uncharged molecules. The signal ratios remained at around 1, showing little difference within each analyte-reference droplet pair and across different glucose concentrations (Supplementary Fig. 8). This is consistent with the fact that the addition of glucose does not change the ionic strength and conductivity of the aqueous solution, supporting our proposition that MRD measures the conductivity of non-magnetic samples.

The leveling off of the signal at high concentrations (Supplementary Fig. 7) might be related to the limited dynamic range of the MRD as well as the fact that the conductivity of salt solutions is not proportional to the concentration due to a decrease in mobility of ions at high concentrations.

#### pH study

To isolate the effect of pH changes on MRD response, a pH gradient was prepared by mixing same concentration of di- and mono-sodium phosphates in different percentages, such that the ionic strength remained the same while the relative amount of hydrogen ions to sodium ions decreased as the pH value increased. It was shown that a more alkaline pH results in higher signal readout (Fig. 3d and Supplementary Fig. 9).

### Magnetic nanoparticles

For magnetic detection, iron oxide particles (Fe_3_O_4_) were used with the sizes of 9.7 nm, 14.8 nm, 16.8 nm and 20.3 nm. Transmission electron microscopy (TEM) images of the samples are shown in Supplementary Fig. 10a. Magnetic characterization was carried out at various temperatures using SQUID. Particles of different sizes possess different saturation magnetization (Supplementary Fig. 10b). Samples with nanoparticles with sizes down to 14.8 nm show a ferromagnetic behavior with a blocking temperature above room temperature. The sample with the smallest particles (9.7 nm diameter) reveals superparamagnetic response with a blocking temperature of about 39 K. Further details on the properties of Fe_3_O_4_ nanoparticles can be found elsewhere^34^.

In addition to the Fe_3_O_4_ nanoparticles, covalently functionalized Cobalt nanoparticles are also used for magnetic detection studies. The core-shell particles consist of about 50 nm in diameter Cobalt core covered by 30 nm thick SiOx shell (TurboBeads Llc). These particles are ferromagnetic at room temperature with saturation magnetization of 158 emu g^−1^. They are dispersed in water by using 2% sodium dodecyl sulfate (SDS) as a surfactant.

### Magnetic detection

As the working principle of the detector is based on monitoring the change of the signal amplitude upon modification of the coils inductance, the most natural is to study droplet trains with encapsulated magnetic particles. As the magnetic nanoparticles traverse the core of the coil, the high permeability 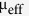
will cause a change in the effective inductance:


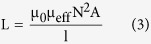


which is detected as a modification of the signal amplitude at constant frequency of the circuit.

As a test object, we fabricated Fe_3_O_4_ nanoparticles with different mean diameters from 10 to 20 nm which were dispersed in deionized water containing 1% sodium dodecyl sulfate (SDS) as a surfactant by sonication for 10 min. Deionized water containing 1% SDS was used in reference droplets. The time evolution of the signal, measured for different concentrations of magnetic nanoparticles in the droplet is shown in Fig. 1c and Supplementary Fig. 11. Two neighboring peaks correspond to the analyte and reference droplets. Higher concentrations of the magnetic nanoparticles inside emulsion droplets lead to a corresponding linear increase of the MRD signal (Fig. 2d). After measuring a wide range of concentrations of magnetic nanoparticles, we determined the detection limit of the device for magnetic particles to be about 10 μg mL^−1^, which lies in the range typically employed for the cell toxicity tests in the laboratory.

As the signal strength is increasing with the concentration of nanoparticles in a droplet, the signal of the detector can be proportional to the total magnetic moment of the droplet or to the total amount of magnetic material in it. To discriminate between these two possibilities, we investigated the response of the detector on measurement of assemblies of Fe_3_O_4_ particles of different size from 10 to 17 nm in diameter. In contrast to smaller nanoparticles, which are superparamagnetic and therefore do not show tendency to agglomeration, larger Fe_3_O_4_ nanoparticles of 17 nm diameter as well as ferromagnetic Co nanoparticles display a certain ferromagnetic response resulting in clustering. Clustering of magnetic particles causes the magnetic moment of nanoparticles to cancel each other, leading to a decrease in the total magnetic moment of the droplet. Nanoparticles encapsulated in the droplets were sequentially analyzed using the resonance detector by recording signal of each sample over 30 min. There was not a major decrease of the signal strength for the samples containing Fe_3_O_4_ nanoparticles (Supplementary Fig. 12). Interestingly, after monitoring over several hours the droplets containing Co nanoparticles of 50 nm diameter, the signal of the MRD starts to decay indicating clustering of the nanoparticles (Supplementary Fig. 13). Agglomeration of the nanoparticles results in the lowering of the total magnetic moment of the droplet. This is reflected in the decrease of the MRD signal. This implies that the detector was sensitive to the total magnetic moment of the material in a droplet.

## Additional Information

**How to cite this article**: Karnaushenko, D. *et al.* Monitoring microbial metabolites using an inductively coupled resonance circuit. *Sci. Rep.* **5**, 12878; doi: 10.1038/srep12878 (2015).

## Supplementary Material

Supplementary Information
