# Supplementary material for: Monitoring microbial metabolites using an inductively coupled resonance circuit
Source: Sci Rep. 2015 Aug 12;5:12878. doi: 10.1038/srep12878 (PMC4533006; doi:10.1038/srep12878)
Supplement: Supplementary Information [file srep12878-s1.pdf]

SUPPLEMENTARY INFORMATION

Monitoring microbial population dynamics using an inductively coupled resonance circuit

D. Karnaushenko[1], L. Baraban[2,*], D. Ye[2], I. Uguz[2], R. G. Mendes[3], M. H. Rümmeli[3], J. A. G. M. de Visser[4], O. G. Schmidt[1,5,6], G. Cuniberti[2,7], D. Makarov[1]

[1] Institute for Integrative Nanosciences, IFW Dresden, Helmholtzstrasse 20, 01069 Dresden, Germany

[2] Max Bergmann Center of Biomaterials, Dresden University of Technology, Budapesterstrasse 27, 01069 Dresden, Germany

[3] Institute for Solid State Research, IFW Dresden, Helmholtzstrasse 20, 01069 Dresden, Germany

[4] Laboratory of Genetics, Wageningen University, 6708PB, Wageningen, The Netherlands

[5] Material Systems for Nanoelectronics, Chemnitz University of Technology, Reichenhainerstrasse 70, D-09107 Chemnitz, Germany

[6] Merge Technologies for Multifunctional Lightweight Structures, Chemnitz University of Technology, Reichenhainer Strasse 70, 09107 Chemnitz, Germany

[7] Center for Advancing Electronics Dresden, TU Dresden, 01062 Dresden, Germany

* Corresponding author: larysa.baraban@nano.tu-dresden.de

1. Characterization of millifluidic resonance detector

1.1 Schematic description of detector unit

An inductive coil with about 70 windings of an isolated copper wire of 100 µm in diameter is winded around a glass tube with an outer diameter of 1 mm. The coil is soldered to a printed circuit board consisting of zero temperature coefficient ceramic capacitors of 10 nF (resulting in a resonant frequency of ~2 MHz) and an operation amplifier OPA602 with a tuned feedback to the LC tank. That board then connected to a second board which has a peak detector and a USB 2.0 microcontroller C8051F342 having built-in analog to digital converter (ADC) (Supplementary Figures 1 & 2).

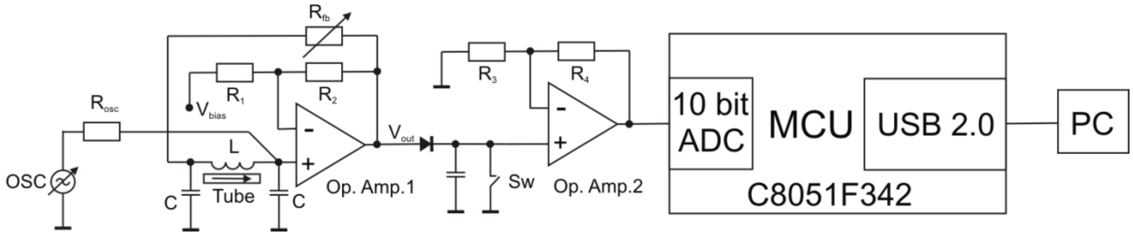

Supplementary Figure 1 | Diagram of the circuit which is used for magnetic resonance detection of droplet content.

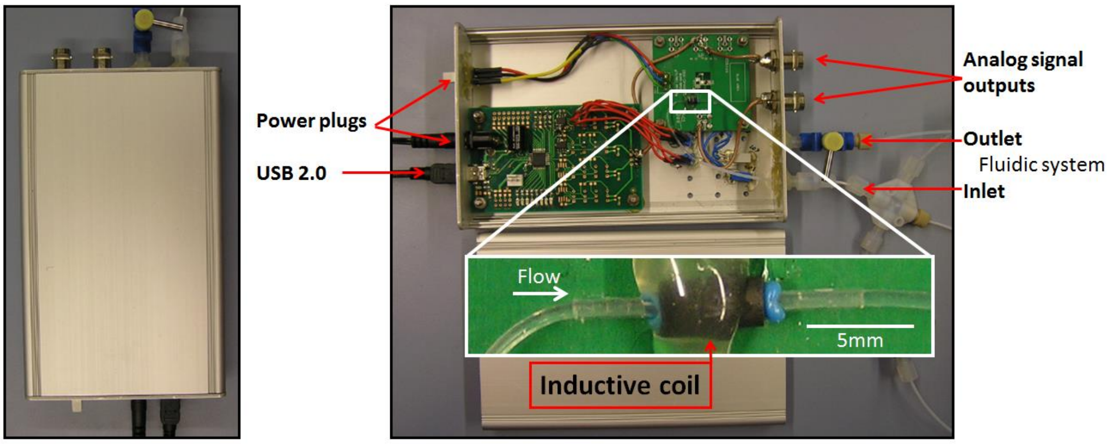

Supplementary Figure 2 | Photos revealing (left panel) the MRD assembled in a commercially available Al case and (right panel) inner structure of the detection module. Inset shows the location of the inductive coil encapsulated in thermo responsive polymeric tube and then fixed by thermoset glue.

The detection circuit (the cascade around the Op. Amp. 1 in Supplementary Figure 1) is represented with the equivalent circuit diagram as shown in Supplementary Figure 3.

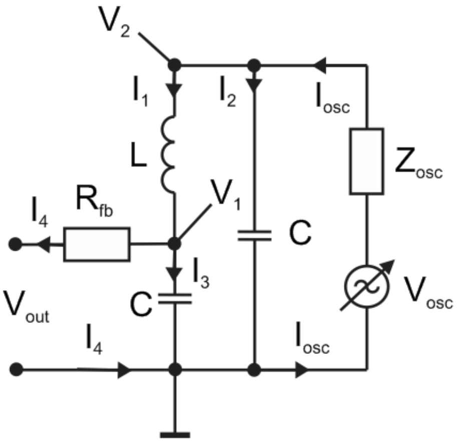

Supplementary Figure 3 | Equivalent circuit diagram of the detection circuit presented in Supplementary Figure 1.

Analysis of this diagram using Kirchhoff law results in the following system of equations for currents and voltages:

$$\begin{cases} I_{osc} = \dfrac{V_{osc} - V_2}{Z_{osc}} \\ I_1 = \dfrac{V_2 - V_1}{Z_L} \\ I_2 = \dfrac{V_2}{Z_C} \\ I_3 = \dfrac{V_1}{Z_C} \\ I_4 = \dfrac{V_1 - V_{out}}{R_{fb}} \\ I_1 + I_2 - I_{osc} = 0; I_3 + I_4 + I_2 - I_{osc} = 0; V_{out} = V_2 KP - V_{bias}(K-1) \end{cases}$$

$$K = \left(1 + \frac{R_2}{R_1}\right);$$
$$P = \cos(\varphi_{amp}) + i\sin(\varphi_{amp})^*$$
$$Z_L = R_L + iX_L$$
$$Z_C = -iX_C$$
$$Z_{osc} = R_{osc} - iX_{osc}$$
$$X_L = \omega L; X_C = \frac{1}{\omega C}; X_{osc} = \frac{1}{\omega C_{osc}};$$
$$\omega = 2\pi f$$

The coefficient P represents the non-inverting phase margin of the Op.Amp.1, which is about $\varphi_{amp} = -100^o$ for the non-inverting configuration for OPA602 at the working frequency of about 2 MHz. Solving this system of equations with respect to the output voltage, V_{out}, the following expression is obtained:

$$\begin{cases} V_{out} = \frac{A}{C}V_{osc} + \frac{B}{C}V_{bias} \\ a = Z_c(Z_c + Z_L) \\ b = Z_c^2 Z_L \\ c = Z_{osc}(Z_L + 2Z_c) \\ A = KP(R_{fb}a + b) \\ B = \left[R_{fb}(a+c) + b + a\right](1-k) \\ C = R_{fb}(a+c) + b + Z_{osc}(a - KPZ_c^2) \end{cases}$$

By tuning the parameters of the circuit, we can achieve almost complete cancellation of the active resistance (about 1 Ohm) of the inductive coil, thus enhancing the quality factor of the LC tank. For the simulations, we use the following values of parameters, which are close to the experimental ones:

$$\begin{cases} C = 10nF; L = 1.266515\mu H; R_L = 1\Omega \\ C_{osc} = 1\mu F; R_{osc} = 100k\Omega \\ R_{fb} = 573.8\Omega; R_1 = 1k\Omega; R_2 = 89k\Omega \\ V_{bias} = 0.7V; V_{osc} = 1.0V; \varphi_{amp} = -100° \end{cases}$$

Supplementary Figure 4 summarizes the result of the simulations. The circuit demonstrates the distinct resonance peak with the full width at half maximum of about 5 Hz, resulting in the quality factor of 10^5. Such a sharp response gives a way to detect a tiny alteration in the coil inductance. If the frequency of the external oscillator is fixed, then the change in the inductance will be detected as a variation in the output amplitude of the circuit. Comparison with the experimental data is provided in Supplementary Figure 4.

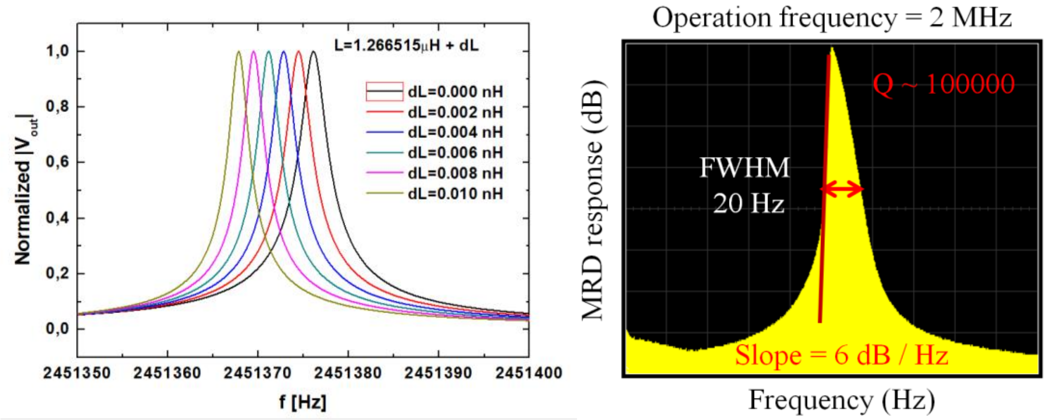

Supplementary Figure 4 | (left) The results of the simulation revealing the normalized absolute value of the output voltage with respect to the frequency of the external oscillator. The presented curves reveal the shift of the resonance peak with the variation of the inductance of the coil. (right) Snap shot from the oscilloscope revealing the resonance peak with full width at half maximum of about 20 Hz when operating at 2 MHz. The MRD response is taken with respect to the noise level of the device.

1.2 Detection of emulsion droplets in fluidic channel by MRD

Water-in-oil emulsion droplets were produced in a cross-junction geometry as a one-dimensional chain, using hydrofluoroether oil (HFE-7500, 3M) as the continuous phase. After formation this chain is pushed towards the detection area. To account for the possible drifts of the signal, a reference droplet (solvent of the sample droplet) is placed next to every sample droplet, so that the signal ratio between the sample and the adjacent reference droplet can provide a more accurate measure of the detector response (Supplementary Figure 5).

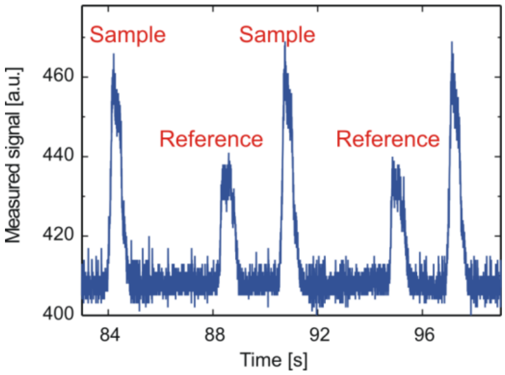

Supplementary Figure 5 | Reading the chain of aqueous droplets of sample (analyte) and reference by the MRD platform.

To minimize the cross-contamination between the two different contents, droplets are produced by pumping the analyte, the oil spacer and the reference sequentially through the cross-junction instead of simultaneous injection, which is the standard way of generating droplets in squeezing regime. Supplementary Figure 6 (left) displays the dependence of the detector signal readout as a function of the droplets size. Obviously, the frequency shift of the resonance circuit increases with an increase of droplet length. It was determined that the signal saturates when the droplet length exceeds the size of the inductive coil (1 mm) and the region of the magnetic stray fields, altogether of about 6 mm for the current detector design. Although, the flow rate was kept constant at 0.25 μL/s in current experiment, the device allows to monitor the droplet flow rate up to 39 μL/s and to detect up to 30-40 droplets per second (limit of the current fluidic circuit) as demonstrated in details in the Supplementary Figure 6 (right panel) and Figure 2(a) of the manuscript. The 10 bit ADC with the max frequency 200 kHz is operating at 800 Hz and can detect safely up to 100 droplets per second, with about 5 points per droplet. However, to achieve this remarkable performance, the fluidic circuitry should be optimized by reducing the number of junctions and interconnects in the fluidic platform. Furthermore, surfactants could be applied to stabilize droplet interfaces.

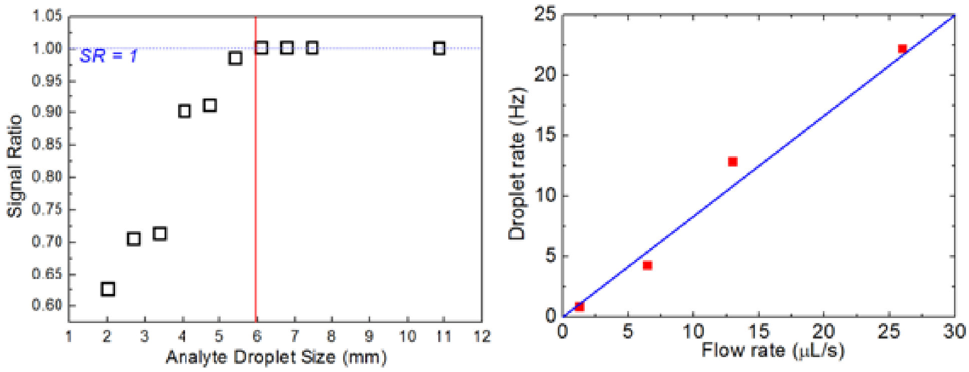

Supplementary Figure 6 | (Left panel) MRD signal vs the size of droplets, pushed through the core of the coil. Signal of the detector does not change if the analyte droplets are larger than about 6 mm (= effective stray field of coil). (Right panel) Detection of the droplets vs droplet flow rate.

2. Characterization of MRD signal ratio (SR)

2.1 MRD signal versus ionic strength of the solution

In order to succeed in monitoring the growth of *E.coli* population over time, the physical quantity measured by the MRD has to be addressed properly, taking into account the resonance circuit specificity and the complexity of the culture medium. To extract the contribution of the bacteria into signal, we experimented with different reference analytes, including solvents of various ionic strength, pH values and magnetic content. Initially, we performed detection of non-magnetic ionic solutions.

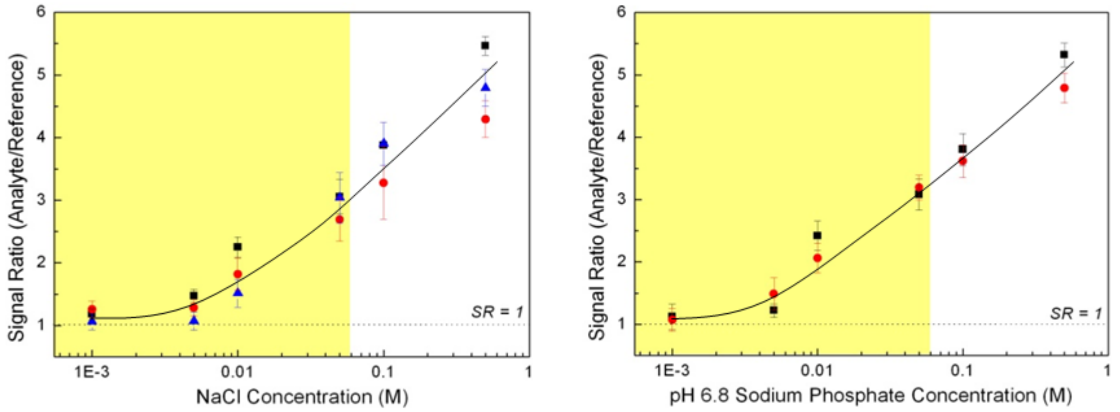

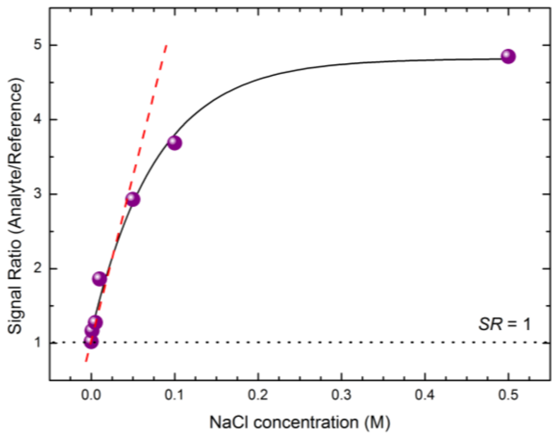

Supplementary Figure 7 | The MRD signal versus the sodium chloride (left panel) and sodium phosphate (right panel) concentrations. Yellow band corresponds to the physiological range of concentrations of salt in the medium. Different symbols correspond to different experiments. (bottom panel) The same data as shown above for the NaCl but plot is in the linear scale (x-axis). The MRD signal is proportional to the concentration of NaCl up to ~0.05 M. Black solid lines are guide to the eye.

Here, the droplets contain aqueous solutions of fully dissociable salts (NaCl, Na phosphates) and the reference droplets contain deionized water. First, we focus on the case of sodium chloride and sodium phosphate, since these substances are typical constituents of the growth medium, helping to maintain its

isotonic balance (Supplementary Figure 7). The MRD signal is proportional to the salt concentration for concentrations of up to about 0.05 M and is the same within the error bars for sodium chloride and sodium phosphate solutions. Considering the proportionality between the concentration of fully dissociable salt solution and its conductivity, this observation indicates proportionality between the power and the conductivity similar to the case of eddy current losses. Hence, the MRD measures the power loss due to induced eddy currents generated within the droplet containing conductive salt solution which is proportional to the ionic strength, or more generally, the conductivity of the droplet content. To cross-check this assumption, we carried out additional tests on glucose as an example of non-ionic and uncharged molecules. The signal ratios remained around 1, showing little difference within each analyte-reference droplet pair and across different glucose concentrations (Supplementary Figure 8). This is consistent with the fact that addition of glucose does not change the ionic strength and conductivity of the aqueous solution, supporting our proposition that the MRD measures conductivity of non-magnetic samples.

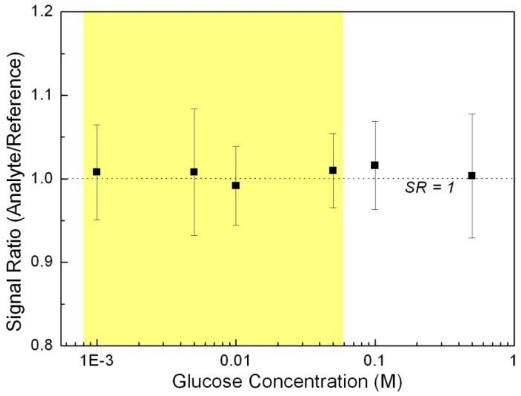

Supplementary Figure 8 | The MRD signal readout at different concentrations of glucose. Yellow field marks the range of glucose concentrations, available in the culture medium.

2.2 MRD signal versus pH values of the solution

To isolate the effect of pH changes on MRD response, a pH gradient was prepared by mixing the same concentration of di- and mono-sodium phosphates in different percentages, such that the ionic strength remained the same while the relative amount of hydrogen ions to sodium ions decreased as the pH value increased. We show that a more alkaline pH results in higher signal readout (Supplementary Figure 9).

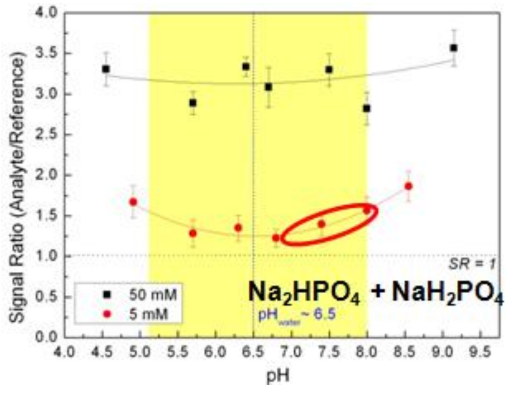

Supplementary Figure 9 | The MRD signal at different pH values of the solutions. Red curve corresponds to the signal measured in a solution with 5 mM; black curve is measured at the salt concentration of 50 mM.

Near the neutral pH, the MRD is not sensitive to the pH change. The signal increases when the pH approaches the edge of the physiological range.

2.3 Detection of magnetic species by MRD

<u>Magnetic nanoparticles</u>

For magnetic detection, iron oxide particles (Fe_3O_4) were used with the sizes of 9.7 nm, 14.8 nm, 16.8 nm and 20.3 nm. Transmission electron microscopy (TEM) images of the samples are shown in Supplementary

Fig. 10a. Magnetic characterization was carried out at various temperature using SQUID-VSM (Quantum design). For these purposes the as-produced samples were dried and small amounts of the powder were weighted before both the magnetization over external field (M vs B) and temperature (M vs T) were conducted. The heating experiments were performed in purpose built equipment with an operating frequency of 120 Hz. The samples were dispersed in an aqueous solution using a tip sonicator with a concentration of 5 mg/mL and measured over 4 min. Particles of different sizes possess different saturation magnetization (Supplementary Figure 10b). Samples with nanoparticles with sizes down to 14.8 nm show a ferromagnetic behavior with a blocking temperature above room temperature. The sample with the smallest particles (9.7 nm diameter) reveals superparamagnetic response with a blocking temperature of about 39 K. Further details on the properties of Fe_3O_4 nanoparticles can be found elsewhere [Mendes 2014].

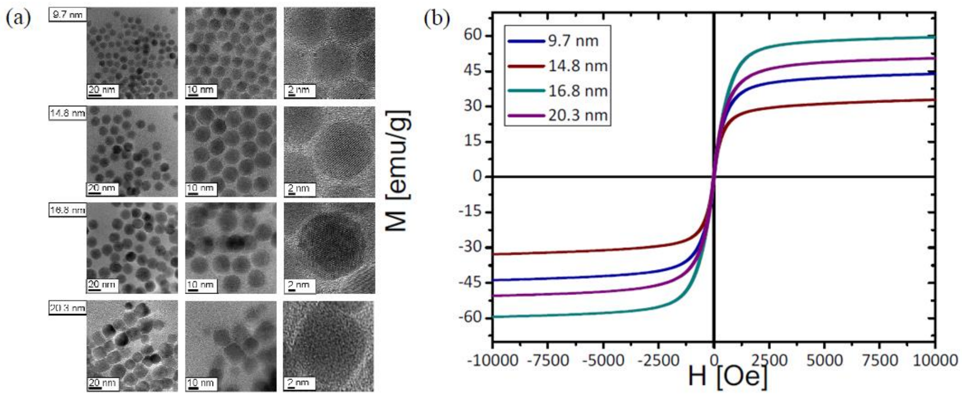

Supplementary Figure 10 | (a) TEM images and (b) Magnetization curves of the Fe_3O_4 nanoparticles for the sizes of 9.7 nm, 14.8 nm, 16.8 nm and 20.3 nm.

In addition to the Fe_3O_4 nanoparticles, covalently functionalized Cobalt nanoparticles are also used for magnetic detection studies. The core-shell particles consist of about 50 nm in diameter Cobalt core covered by 30 nm thick SiOx shell (TurboBeads Llc). These particles are ferromagnetic at room temperature with saturation magnetization of 158 emu/g. They are dispersed in water by using 2% sodium dodecyl sulfate (SDS) as a surfactant.

Magnetic detection

As the working principle of the detector is based on monitoring the change of the signal amplitude upon modification of the inductance of the coil, the most natural is to study droplet trains with encapsulated magnetic particles. As the magnetic nanoparticles went through the core of the coil, the high permeability μ_{eff} will cause a change in the effective inductance:

$$L = \frac{\mu_0 \, \mu_{eff} \, N^2 A}{l}$$

which is detected as a modification of the signal amplitude at constant frequency of the circuit.

As a test object, we fabricated Fe_3O_4 nanoparticles with different diameters from 10 to 20 nm which were dispersed in deionized water containing 1% of sodium dodecyl sulfate (SDS) as a surfactant by sonication for 10 minutes. Deionized water containing 2% SDS was used in reference droplets. The time evolution of the measured signal; concentration of magnetic particle in the droplet is a parameter, which was modified (Supplementary Fig. 11). Two neighboring peaks correspond to the analyte and reference droplets. Increase of the concentration of magnetic particles in a droplet results in a linear increase of the detected signal. The detection limit of the device is about 0.02 mg/ml. This number is in the range usually employed for the cell toxicity tests in the laboratory.

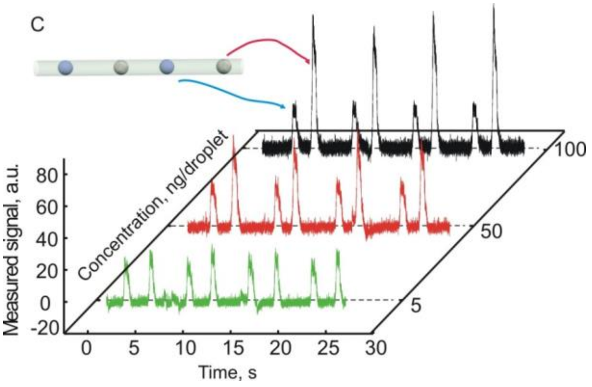

Supplementary Figure 11 | The measured signal (raw data), showing the effect of magnetic nanoparticle concentration on the MRD response.

As the signal strength is increased with the concentration of nanoparticles in a droplet, the signal of the detector can be proportional to the total magnetic moment of the droplet or to the total amount of magnetic material in it. To discriminate between these two possibilities, we investigated the response of the detector on measurements of assemblies of Fe_3O_4 particles of different sizes from 10 to 17 nm in diameter. In contrast to smaller nanoparticles, which are superparamagnetic and therefore do not show tendency to agglomeration, larger Fe_3O_4 nanoparticles of 17 nm diameter as well as ferromagnetic Co nanoparticles display a certain ferromagnetic response resulting in clustering (Supplementary Figures 12 & 13). Clustering of magnetic particles causes the magnetic moment of nanoparticles to cancel each other, leading to a decrease in the total magnetic moment of the droplet. Nanoparticles encapsulated in the droplets were sequentially analyzed using the resonance detector by recording the signal of each sample over 30 minute. Although particles agglomerate, there was not a major decrease of the signal strength for the samples containing Fe_3O_4 nanoparticles.

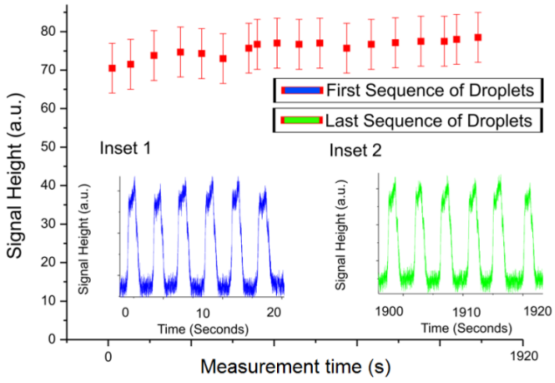

Supplementary Figure 12 | Time evolution of the signal ratio for Fe_3O_4 nanoparticles with a 17 nm diameter. The insets correspond to the first and last sequence of droplets.

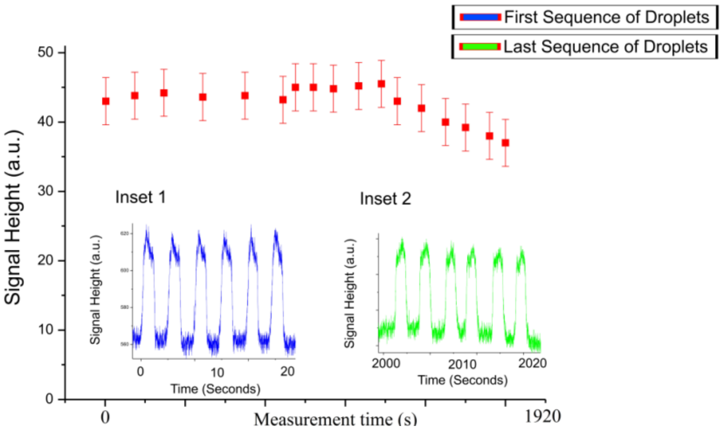

Supplementary Figure 13 | Time evolution of the signal ratio for Co nanoparticles with a 50 nm diameter. The insets correspond to the first and last sequence of droplets.

Interestingly, after monitoring over several hours the droplets containing Co nanoparticles of 50 nm diameter, the signal of the MRD starts to decay indicating clustering of the nanoparticles (Supplementary

Figure 13). Agglomeration of the nanoparticles results in the lowering of the total magnetic moment of the droplet. This is reflected in the decrease of the MRD signal.

3. Measurements of bacterial culture

3.1 Calibration measurements: cell count *vs* optical density OD_{600}

In order to relate the measurements of optical density, OD600, to the MRD signal and to access the number of cells per milliliter, we calibrated OD_{600} by counting bacteria using micro-grid and dry mass determination techniques. The summary over the calibration is presented in Supplementary Figure 14.

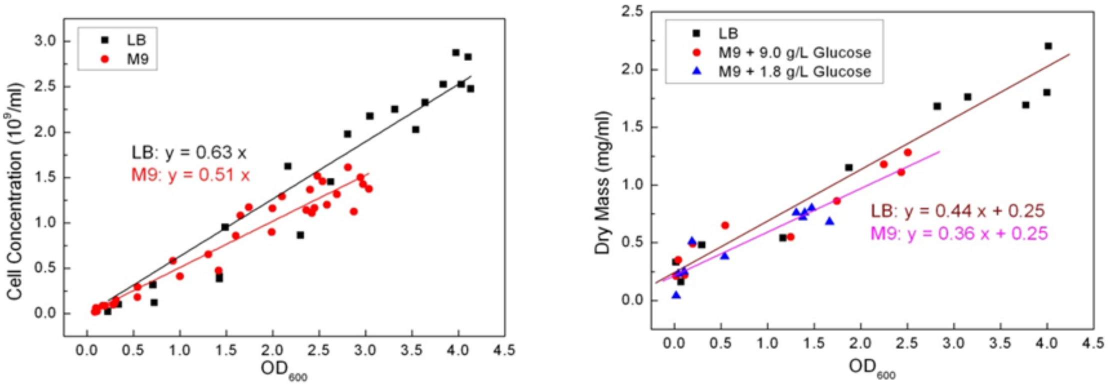

Supplementary Figure 14 | (left panel) Cell count (grid) versus optical density. (right panel) Dry mass calibration versus optical density. Measurements are performed for *E.coli* grown in Luria Bertani broth (LB) and minimum salts M9 media.

3.2 Measurement of ammonium concentration in *E.coli* culture medium

Ammonium is a prevalent salt present in biological systems. It is an important source of nitrogen, which is crucial for the synthesis of proteins and nucleic acids in cells. It also constitutes a major part of cellular wastes. The concentration of ammonium in the growing culture was determined by means of a colorimetric assay using test kits.As shown in Supplementary Figure 15, the concentration of ammonium decreases for M9 minimal medium, in which NH_4Cl is their only source of nitrogen. Ammonium was consumed, and the amount decreases proportionally to the cell concentration. For LB medium, the concentration increases as a result of assimilation of proteins in tryptone as the carbon source shortly after the start of incubation. Deamination of the amino acids produced ammonium that exceeds the need of the cells. As a result, concentration of ammonium increases roughly proportionally to the cell concentration.

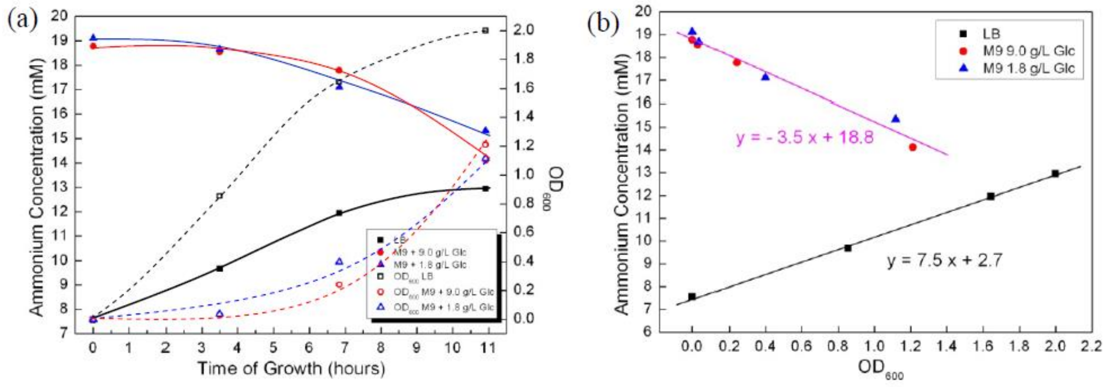

Supplementary Figure 15 | (left panel) Detection of ammonia concentration in the medium of a growing culture in LB broth and M9. Classical growth curve (opened symbols and dotted line) were plotted on the same time scale. (right panel) Ammonium concentration versus optical density (i.e. cell concentration).

3.3 Measurement of acetate concentration in *E.coli* culture medium

Acetate is one of the major waste products of anaerobic respiration and the entry point of Krebs cycle. The amount of acetate in the growing culture was evaluated by gas chromatography (GC) HP 6890 with a polyethylene glycol-capillary column HPINNOWax 19091N-233 (30 m x 0.25 mm x 0.5 μm, HP, FID-detector). 50 μl of culture medium with bacterial cells removed was mixed with 50 μl of 0.2 M phosphoric acid to improve volatility and 100 μl of 10 g/L n-pentanol in water as the internal standard. The concentration of compound in the sample was calculated as the peak area at a characteristic retention time in relation to the peak area of the internal standard.

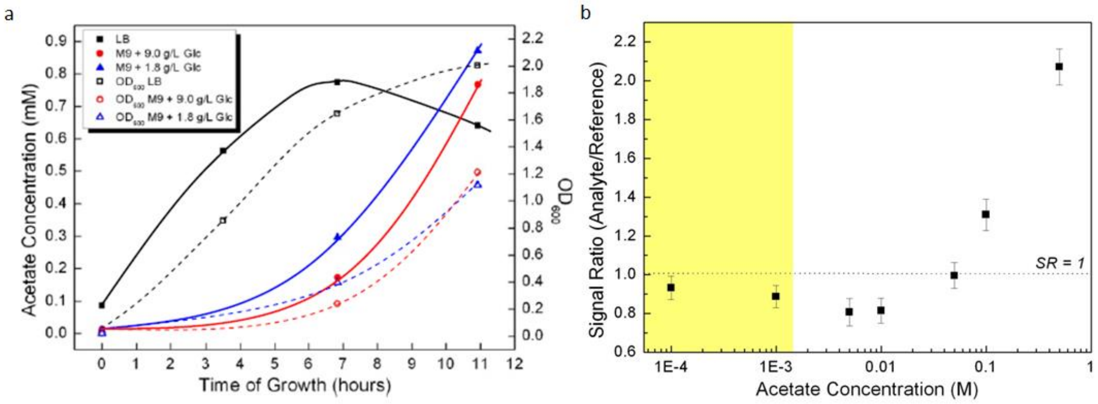

Supplementary Figure 16 | (left panel) Detection of acetate concentration in the medium of a growing culture in LB broth and M9. (right panel) MRD signal at different acetate concentrations in the solutions.
